# Positive anticipated affective reactions increase pro-environmental behavior

**DOI:** 10.1016/j.isci.2025.112389

**Published:** 2025-04-08

**Authors:** Camilla Strömbäck, Per A. Andersson, Erkin Asutay, Hulda Karlsson-Larsson, Daniel Västfjäll

**Affiliations:** 1 JEDI Lab, Division of Economics, Department of Management and Engineering, Linköping University, Linköping, Sweden; 2 JEDI Lab, Division of Psychology, Department of Behavioral Sciences and Learning, Linköping University, Linköping, Sweden; 3 Decision Research, Eugene, OR, USA

**Keywords:** Environmental science, Environmental policy, Social sciences, Interdisciplinary application studies, Psychology

## Abstract

With households accounting for 75% of global carbon emission, we need to find ways to motivate people to increase their climate change mitigating behaviors. Affective reactions have been linked to pro-environmental behaviors (PEBs), but it is unclear if positive (warm glow or hope) or negative (worry or hopelessness) is more impactful in motivating action. In this pre-registered study we used a unique longitudinal dataset from a Swedish sample to reveal that positive affect predicts more engagement in PEBs, and negative affect predicts less engagement in PEBs. A mediation model also suggests that anticipated (i.e., expected feelings of) positive affect is related to past pro-environmental engagement and could partly predict future PEBs. Hence, affective reactions may be one way to increase intrinsic motivation for a more sustainable lifestyle.

## Highlights

• Participants rated engagement in in pro-environmental behaviors (PEBs)
• Anticipated affect and behavioral intentions in Time 1 predict PEBs in Time 2
• Anticipated positive affect predicts more engagement in PEBs
• Anticipated negative affect predicts less engagement in PEBs

## Graphical abstract

**Figure:**
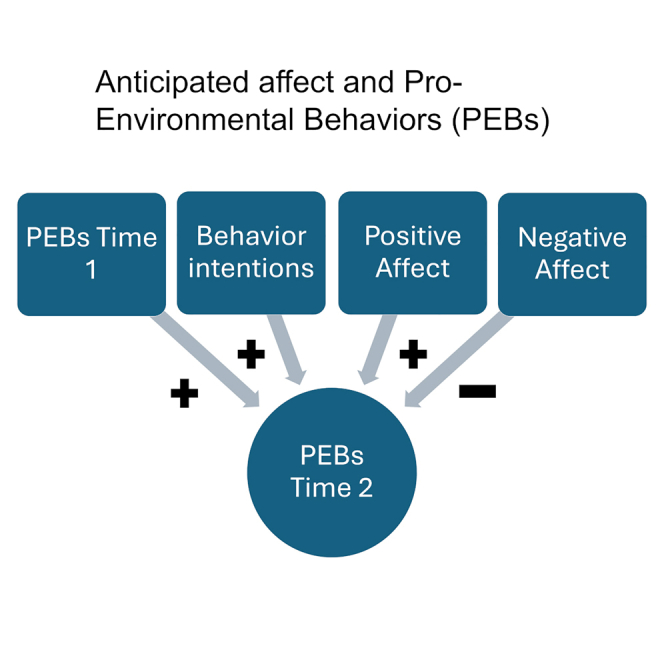

## Introduction

Year 2024 was the warmest year on record with near-surface temperatures of 1.55°C above pre-industrial levels,^1^ which is close to those well below 2°C that was agreed upon in the Paris Agreement.^2^ As the temperature on Earth is rising and leading to non-reversable damage, fighting climate change is one of the greatest and most important challenges for humanity to date. As households account for almost 75% of global carbon emissions,^3^ one critical point is to study and understand the drivers for individuals’ everyday choices and consumer behaviors. Emotions have been identified as a central component when addressing climate change and promoting individuals’ sustainable behavior. Several calls have recently been made to investigate the role of emotions, specifically aspects such as empirical studies of anticipated emotions and the integration of such empirical findings and theory.^4^^,^^5^^,^^6^

Emotion and affect have several connections to climate change and pro-environmental behaviors (PEBs). To begin with, there is clear evidence indicating a relationship between emotions and climate change related psychological outcomes, such as the perceived risk of climate change,^7^^,^^8^^,^^9^ pro-environmental intention,^9^^,^^10^ adaptation behaviors,^11^ belief in climate change,^12^ and policy support.^13^^,^^14^ As a result of this relationship, intervention strategies utilizing both positive and negative emotions are beginning to be explored.^15^^,^^16^ However, there is still great uncertainty regarding when and for whom positive and negative emotions respectively are effective for increasing climate change mitigative behaviors.^17^ Indeed, there is some evidence indicating that using positively valenced stimuli (e.g., emphasizing solutions to climate change) help fuel important psychological antecedents for PEBs,^18^^,^^19^ while other evidence conversely suggests that negatively valenced stimuli (e.g., climate change consequences) would be more powerful in fueling mitigative action.^18^

Beyond the uncertain impact of positive and negative valenced stimuli, there is also a time component when studying how affect influences decisions. For many behaviors there is a time gap between the decision to act, or not to act, and the experienced affect from the behavior.^20^ As a consequence, the decision maker may incorporate the anticipated affect, i.e., “the prospect of feeling positive or negative emotions, e.g., exhilaration or regret, after performing or not performing a behavior”,^21^ into his or her decision.^22^ If the behavior happens repeatedly, the emotions and affective experiences we have in response to the behavior, may affect how we expect to feel, e.g., the anticipated affect, from that stimulus in the future, leaving an affective residue that impacts similar situations in the future.^23^ As the time component is central, there is a need for studies with repeated measures across time.

Several previous studies have found a link between anticipated affect or anticipated warm glow and behavior intentions. For example, both positive and negative anticipated emotions from acting against climate change correlated with intentions to act against climate change.^24^ The self-reported intentions to purchase an electric car correlated positively with positive anticipated emotions and negatively with negative anticipated emotions.^25^ Also, Jerit et al.^26^ found a causal link between warm glow and several PEBs. However, although behavioral intentions can be a good proxy for actual behavior,^27^ there is an observed intention-behavior gap for PEBs.^28^^,^^29^ The intention-behavior gap creates a limitation in much of previous research. Therefore, it is of great interest to study not only behavioral intentions but also self-reported behavior.

For example, anticipated warm glow from acting pro-environmentally has been found to correlate with several self-reported PEBs.^30^^,^^31^ In van der Linden,^31^ the focus was on anticipated warm glow from acting green in general, which predicted low-cost self-reported PEBs comparable to the behaviors in this present study. This relationship was partly mediated by behavioral intentions. However, the evidence is limited by their focus on a general measurement of anticipated warm glow from acting green, rather than anticipated affect connected to the specific behaviors that were investigated. Additionally, by focusing solely on warm glow, they include no measure of negative affect in their study. PEBs and consumer behaviors can vary greatly in perceived difficulty as well as associated costs, making it likely that affective associations will vary between different specific behaviors, as well as having both a negative and a positive dimension. Thus, building measurements on affect toward specific behaviors, rather than toward a domain of behavior, may produce more precise estimates.

Within any domain of behaviors, individuals may feel a mix of positive and negative emotions in anticipation of each specific behavior. The affect heuristic^32^^,^^33^ explains how people often rely on feelings regarding “goodness” or “badness” when making judgments. Based on prior experiences and associations, people anticipate how they will feel when enacting a behavior. Positive anticipated affect motivates a person to try to reproduce the feeling, while negative anticipated affect has the opposite effect. In this way affect motivates behavior.^34^ While previous research on the affect heuristic has explored how people perceive climate change and its associated risks,^34^ it can also serve as a valuable tool for understanding everyday behaviors aimed at mitigating climate change. Here, we use the affect heuristic as a starting point to investigate how people view PEBs, which involves measuring both positive and negative affect related to the PEBs.

To investigate this, we propose a longitudinal design as longitudinal studies investigating anticipated affect and behavior are lacking in this area with few exceptions.^30^^,^^31^ We specifically make novel contributions to the literature in the following ways: First, to get a better understanding of the complex relation between anticipated affect and behaviors, we include separate measures for positive and negative anticipated affect related to the specific behaviors. The reason behind this is that although engaging in PEBs often is perceived as desirable,^35^ many PEBs are also seen as a sacrifice or a burden^36^ or not in line with the social norm, e.g., choosing a vegan diet or choosing not to fly.^37^ Due to this duality, we believe that the same behavior can simultaneously trigger both positive and negative affect in a person. Second, the longitudinal design allows us to measure PEBs at T1 and T2, which enables us to include previous behavior as a predictor in our models and to investigate the relation between anticipated affect and behavior at different points in time. The idea behind this is that engagement in a behavior which induces more positive and less negative affect may increase more positive and less anticipated negative affect for that behavior leading to stronger intention and future engagement.^6^ Third, by focusing on affect rather than warm glow or specific emotions, this study contributes to a better understanding of the emotional drivers behind PEBs. It is likely that several everyday PEBs do not necessarily generate a specific discrete positive and/or negative emotion, but rather a faint whisper of emotion,^4^^,^^20^ which can be hard to specify. Measures of positive and negative affect could pick up such a faint whisper that measures of discrete emotions may miss. Thus, in the current study, we investigate how anticipated positive *and* negative affect from engaging in a specific everyday PEB predicts the degree of engagement in that specific behavior during the following four weeks. By having time pass between the ratings of affect and the measurement of behaviors, we ensure that we can capture anticipated affect, rather than only capturing concurrent affect.

The present study thus addresses three gaps in the literature: It uses measurements at multiple time points, captures self-reported behavior rather than behavioral intention, and focuses on positive and negative affect anticipated in relation to the relevant behaviors. Based on previous research on emotions and PEBs, our general hypothesis is that participants who anticipate a specific PEB to generate positive affect are more likely to engage in that behavior and have stronger intentions to implement it. For negative affect we predict the opposite relation. Thus, we propose the following hypotheses.

H1: There is a positive correlation between positive anticipated affect connected to a specific PEB and the likelihood to engage in that behavior during the following weeks.
H2: There is a negative correlation between negative anticipated affect connected to a specific PEB and the likelihood to engage in that behavior during the following weeks.

## Results

### Descriptive results

**Figure 1:**
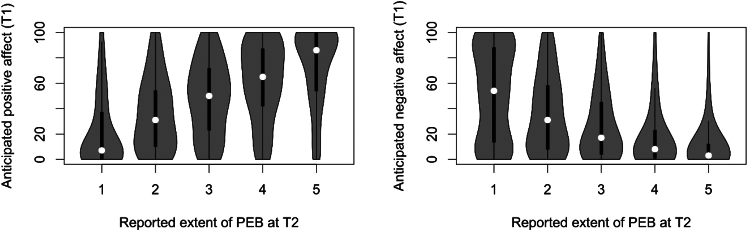
The relationship between pro-environmental behavior reported at Time 2 and anticipated positive and negative affect reported four weeks earlier (Time 1) White dots represent the median values, the black rectangles the interquartile range, and the gray areas illustrate distribution of values.

We first studied the average anticipated positive and negative affect, pro-environmental intention, pro-environmental engagement (reported at Time 1 and Time 2) for each behavior (Table 1), which suggests a pattern that participants anticipated to have more positive and less negative affect for the behaviors that are undertaken to a larger extent. Spearman rank-order correlations indicates that PEB reported at Time 2 was significantly correlated with anticipated positive (*Spearman’s rho = 0.49, p < 0.001*) and negative affect (*Spearman’s rho = −0.41, p < 0.001*) reported at Time 1 (Figure 1).

**Table 1:** Mean and standard deviation presented for the measures anticipated affect, intentions to engage in pro-environmental behaviors and self-reported engagement with pro-environmental behaviors

Behavior	Positive affect (T1)	Negative affect (T1)	Intention (T1)	PEB (T1)	PEB (T2)
Eating less meat	38.7 (28.3)	35.3 (28.6)	2.5 (1.0)	2.5 (1.0)	2.6 (1.1)
Having a low indoor temperature	40.3 (28.6)	37.3 (28.8)	3.1 (1.2)	3.0 (1.2)	3.1 (1.2)
Reducing car transport	56.4 (25.4)	26.5 (23.3)	3.0 (1.2)	3.0 (1.3)	3.1 (1.3)
Taking 5-min showers, or shorter	55.1 (27.0)	21.4 (22.8)	3.4 (1.1)	3.3 (1.2)	3.4 (1.1)
Hang drying laundry	59.2 (25.7)	18.0 (20.3)	3.8 (1.1)	3.7 (1.2)	3.8 (1.1)
Turning-off unnecessary lights	60.7 (25.8)	13.6 (18.7)	3.7 (1.0)	3.6 (1.0)	3.8 (0.9)
Waste sorting	68.8 (22.3)	14.1 (17.2)	4.4 (0.9)	4.4 (0.9)	4.5 (0.8)

Anticipated negative and positive affect were assessed on a 0–100 scale. Intention and PEB are assessed on 5-point scales (1–5). Within-subject standard deviations are reported in parentheses. PEBs were measured at both Time (T1) and Time 2 (T2).

### Anticipated affect predicts pro-environmental behavior

We next ran linear mixed models to study whether anticipated positive and negative affect from carrying out a sustainable behavior can predict the engagement with the behavior during the four weeks following the survey. The model included fixed effects of anticipated positive and negative affect as well as random intercepts at both individual and behavior levels (see Model 1, Table 2). We found that anticipation of greater positive affect and lower negative affect predicted an increased engagement with PEBs during the following four weeks with significant coefficient estimates. We next controlled for past pro-environmental behavior (PEB at Time 1) and intention to engage in sustainable behaviors, both reported at Time 1, by including them as fixed effects in the model (see Model 2 in Table 2). Importantly, the coefficient estimates for anticipated positive and negative affect were significant, albeit substantially reduced, in predicting pro-environmental behavior reported at Time 2. In addition, both intention and previous PEB were positively associated with behavior reported four weeks later.

**Table 2:** Standardized coefficient estimates for the fixed effects model predicting pro-environmental behavior reported at Time 2

Predictors	Model 1	Model 2A	Model 2B	Model 3
	St.Beta [95% CI]			St.Beta [95% CI]
Anticipated positive affect (T1)	0.32^a^ [0.30, 0.34]	0.08^a^ [0.06, 0.1]	0.08^a^ [0.06, 0.09]	0.05^a^ [0.03, 0.07]
Anticipated negative affect (T1)	−0.22^a^ [−0.25, −0.20]	−0.05^a^ [−0.07, −0.03]	−0.06^a^ [−0.08, −0.04]	−0.04^a^ [−0.05, −0.02]
PEB (T1)			0.67^a^ [0.63, 0.7]	0.42^a^ [0.36, 0.47]
Intention (T1)		0.66^a^ [0.61, 0.71]		0.32^a^ [0.27, 0.37]
Age	0.01 [−0.01, 0.03]	0.01 [−0.01, 0.03]	0.01 [−0.01, 0.03]	0.01 [−0.01, 0.02]
Female	0.05^b^ [0.01, 0.10]	0.01 [−0.02, 0.05]	0.02 [−0.01, 0.05]	0.01 [−0.02, 0.05]
Education	0.04^a^ [0.02, 0.07]	0.02 [−0.01, 0.03]	0.02^b^ [0.01, 0.04]	0.02 [0.00, 0.03]
Income	−0.04^a^ [−0.07, −0.02]	−0.03^a^ [−0.05, −0.01]	−0.03^a^ [−0.04, −0.01]	−0.02^c^ [−0.04, −0.01]
(Intercept)	−0.03 [−0.27, 0.22]	0.01 [−0.08, 0.1]	0.01 [−0.09, 0.1]	0.01 [−0.07, 0.08]
Marginal R^2^/Conditional R^2^	0.24/0.41	0.57/0.64	0.59/0.65	0.62/0.68

See also Tables S1 and S2.

a *p* < 0.001.

b *p* < 0.05.

c *p* < 0.01.

### Exploratory analysis

**Figure 2:**
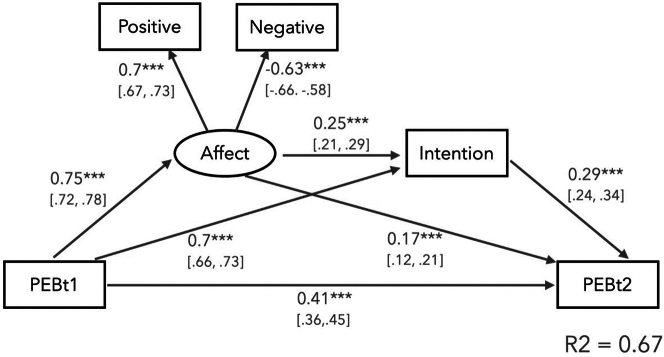
The theoretical model showing all indirect and direct paths from PEB reported at time 1 to time 2 via anticipated affect and pro-environmental intentions Standardized regression coefficients and 95% confidence intervals (in brackets) are shown for each path. ∗∗∗*p* < 0.001.

We ran a mediation model to test whether past pro-environmental engagement is associated with the anticipation of more positive and less negative affect, which in turn enhances both intentions and future pro-environmental engagement (chi-sq(2) = 4.9, *p* = 0.08, CFI = 0.99, TLI = 0.99, RMSEA = 0.015, SRMR = 0.002). The model explains 67% of the variance in pro-environmental engagement reported at Time 2 (Figure 2) and all parameter estimates were significant (ps < 0.001). These exploratory results suggest that anticipated positive affect are related to past pro-environmental engagement and could partly predict future PEB. Although this finding suggests that affect can both be an antecedent and a consequence of pro-environmental actions, it should be taken with caution because we cannot lay out a definitive causal structure with the current data.

## Discussion

Drawing on a unique, longitudinal Swedish dataset, this study demonstrates that participants who anticipated a specific PEB to generate positive affect were more likely to engage in that behavior and had stronger intentions to implement it. The opposite was found for negative affect; if participants anticipated negative affect from completing a specific PEB, they were less likely to engage in the behavior and had lower intentions to implement it. Hence, the results are of interest for future studies of affective paternalism which implies that affect can be used on a societal level to encourage, among other things, prosocial and pro-environmental behaviors.^38^

Understanding the link between behavior and emotion is important for several reasons. For example, to build better campaigns promoting PEBs by utilizing emotions, and to be able to predict how PEBs can be sustained over time, we first need to understand how affect and emotions influence the decisions we make. As campaigns to implement behavioral change have shown to have limited impact in the long run and often need regular reinforcements,^6^ using emotions to generate intrinsic motivation for change toward a more sustainable lifestyle could be one way forward. For that reason, the theory of a self-reinforcing feedback loop is appealing.^6^ Building on the ideas formalized by Baumeister et al.,^23^ the theory of an affective feedback loop assumes that affect and emotions caused by previous choices will influence our future choices when facing a similar situation. Hence, experiencing positive affect when engaging in PEBs will increase the likelihood of anticipating positive affect from performing the behavior in the future, which will lead to an increased likelihood of repeating the behavior.^6^ An affective feedback loop could be self-reinforcing and thereby generate more sustainable habits than behavior changes driven by more temporary motives.

An affective feedback loop would imply that campaigns boosting positive affect from engaging in PEBs could be successful. However, more empirical studies of the existence of an affective feedback loop are needed.^4^^,^^6^^,^^39^ The results of the current study could be seen as a first step of such an empirical test as it shows that anticipated affect is associated with future behaviors. Also, our mediation model, while exploratory in nature, illustrates that behavior at an earlier point in time could influence behavior later through anticipated affect and intention. For a more complete model of the affective feedback loop, one would ideally also measure experienced affect after performing the behavior. Further, one would need to track these processes over time, not just as a single iteration, especially as previous studies have found that it is difficult to anticipate one’s future emotions^40^ and also to correctly recall one’s experienced emotions.^41^ As the theory of the affective feedback loop builds on the assumption that we can both accurately forecast our future affect and correctly recall our experienced affect when facing a similar decision again, further research is needed on how experienced affect influences affective forecasting ability and future behaviors. As people generally are poor at judging the effectiveness of their climate mitigation behaviors, and may be more led by their affect,^42^ future studies should also investigate if there is a risk of people getting stuck in affect-driven self-reinforcing emotional loops leading to engagement in inefficient PEBs. Additionally, future studies should investigate different types of PEBs as the effect of anticipated affect on future behaviors may differ between high-cost and low-cost behaviors (van der Linden, 2018). In this study we did not make such a distinction. Apart from the obvious risk of individuals categorizing the same behavior differently, the behaviors often considered as high-cost behaviors happen less often —sometimes only once— which make them harder to investigate in the setting we chose. Instead, we focused on PEBs which occur frequently and could be implemented by almost everyone.

In summary, the results of this study implied a relationship between anticipated affect and engagement in various PEBs. Hence, policy makers should consider how to boost positive affect from sustainable behaviors and mitigate the negative affect that could be generated by the same behaviors to spread a more sustainable lifestyle and increase the likelihood to meet the goals of the Paris Agreement.

### Limitations of the study

One possible limitation of the current study is that we only measure behavior intentions and self-reported behaviors rather than observing behaviors in the field. Self-reported data have several limitations, including social desirability bias and difficulties to rate how often one engages in a behavior.^43^ Another limitation is that the current study focuses on everyday behaviors and does not include behaviors occurring less frequently, such as major investments.

## Resource availability

### Lead contact

Further information and requests for resources should be directed to the lead contact: Camilla Strömbäck, e-mail: camilla.stromback@liu.se.

### Materials availability

The study materials are included in the supplementary materials available at: https://doi.org/10.17632/847bvc7g38.1.

### Data and code availability

• The dataset to perform the statistical analysis is deposited on OSF (https://osf.io) and is publicly available as of the date of publication (https://osf.io/zxqvn/).
• The code used for statistical analysis is deposited on OSF (https://osf.io) and is publicly available as of the date of publication (https://osf.io/zxqvn/).
• The original survey questions are deposited on OSF (https://osf.io) and are publicly available as of the date of publication (https://osf.io/zxqvn/).

## Acknowledgments

This research was funded by the 10.13039/501100004527Swedish Energy Agency (10.13039/501100004527Energimyndigheten) grant No. P2023-00210 and grant No. P2024-00782 as well as 10.13039/501100011898Marianne and Marcus Wallenberg Foundation grant No. KAW 2023.0289.

## Author contributions

C.S., P.A.A., E.A., H.K.-L., and D.V. developed the study concept and design. C.S. and P.A.A. collected the data and E.A. analyzed the results. All authors contributed to the interpretation of the data. C.S., P.A.A., H.K.-L., and E.A. wrote the first draft of the paper. All authors provided revisions and approved the final version.

## Declaration of interests

The authors declare no competing interests.

## STAR★Methods

### Key resources table

**Table:** 

REAGENT or RESOURCE	SOURCE	IDENTIFIER
**Deposited data**		

Survey data	This study	Mendeley data: https://doi.org/10.17632/847bvc7g38.1,^44^

**Software and algorithms**		

R Project for statistical computing	http://www.r-project.org/	RRID:SCR_001905
R package: lme4	https://cran.r-project.org/web/packages/lme4/index.html	RRID:SCR_015654
R package: lmerTest	http://CRAN.R-project.org/package=lmerTest	RRID:SCR_015656
R package: tidyverse	https://CRAN.R-project.org/package=tidyverse	RRID:SCR_019186
R package: lavaan	https://cran.r-project.org/web/packages/lavaan/index.html	RRID: SCR_019107
R package: lavaanPlot	https://cran.r-project.org/web/packages/lavaanPlot/index.html	RRID: SCR_021123
R package: sjPlot	https://cran.r-project.org/web/packages/sjPlot/index.html	RRID: SCR_021124
Qualtrics Survey Platform	https://www.qualtrics.com/research-core/survey-software/	RRID:SCR_016728

### Experimental model and study participant details

#### Participants

Participants were recruited from a Swedish panel company (PFM Research) that provided a demographically representative sample of the Swedish population. Data was collected in two waves, here referred to as Time 1 and Time 2. As we expected some attrition between the first and second wave of data collections 1605 participants with complete data were recruited at Time 1, with the aim of having 1000 of these coming back at Time 2. Participants failing an attention check question in Time 1 were not counted toward our aim of 1600 participants (407 participants, about 20% of those starting the survey in total, failed the attention check and were stopped from continuing). Data collection at Time 2 stopped at 1005 participants, with 10 being excluded from data analysis due to taking the survey multiple times. After exclusion, the final sample used in the analyses consisted of 995 participants (50.1% women, mean age = 48.8, *SD* age = 15.2) for this study. Before starting the survey, the participants gave informed consent, both at Time 1 and Time 2. Upon completing the survey, the participants were paid SEK 55 (about USD 5.3) for their participation.

We consider a Swedish sample relevant for the current research aims, since the human population that needs to change their lifestyle and consumer choices the most is the citizens in the Global North, which includes Sweden. Furthermore, as we were familiar with conditions in Sweden, this allowed us to select appropriate study materials. The research was conducted in accordance with relevant guidelines and regulations, including the Declaration of Helsinki. In accordance with Swedish law, no formal ethical review is needed when the study does not involve aspect such as harm caused or collection of sensitive personal information.

### Method details

#### Procedure

The data for this study was collected as part of a larger survey. Here we will focus on the variables relevant for this particular study, while describing the general procedure and link to the full materials (See supplementary materials). The study was preregistered (https://osf.io/fja4p/?view_only=5fedd250a25842948b7d5f8433c9301d) before data collection. For this study, data was collected in two waves, here referred to as Time 1 and Time 2. The time between the waves was approximately four weeks.

At Time 1 participants began by filling in demographic information including gender, age, education, household income, the number of household members, whether their living space was rented or not, and if so, what was included in the rent. The demographic questions were followed by two separate blocks of questions, in randomized order. The first of these blocks asked about their anticipated positive and negative affect in response to carrying out seven different PEBs (see details below). The second block involved questions regarding affective reactions to participants’ own energy bill, climate change, and the current state of society (not included in this manuscript). Following these two blocks, participants answered questions about their behavioral intentions regarding the seven PEBs. The participants also completed additional measures not of interest to the current work; these measures can be found in our preregistration. Toward the end of the survey, participants answered questions regarding their current engagement in each of the seven PEBs. In total the median time to complete the survey for Time 1 was 17 min.

At Time 2, only participants who had completed the survey at Time 1 were invited to participate and were paid SEK 40 (about USD 3.9) for their participation. This survey started by asking participants about their engagement in the seven PEBs during the previous four weeks since completing the survey at Time1. Thereafter, they completed additional measures that are not relevant for the current study. The median time to complete the survey at Time 2 was 5 min.

#### Measurements and materials

##### Anticipated affect regarding PEBs

Participants were asked “How strong positive [negative] emotions do you think you would experience when you …” followed by the relevant behavior. The seven behaviors were: (i) take showers that last for less than 5 min, (ii) walk, bike, or use public transportation instead of driving, (iii) turn the lights off when leaving a room, (iv) choose another source of protein than animal-based meat, (v) recycle household waste, (vi) hang-dry clothes instead of tumble-drying them, and (vii) have an indoor temperature of 20°C or lower. They then rated anticipated positive affect on a slider scale (from 0 to 100) as well as negative affect (from 0 to 100). Positive and negative affect were asked about and rated independently of each other.

##### Intentions to engage in PEBs

Participants reported their behavior intentions for the same seven PEBs of interest in this study on a scale from 1 = very unlikely to 5 = extremely likely.

##### Engagement in PEBs at time 1

The respondents report how often they had engaged in the same seven behaviors during the last four weeks on a scale from 1 = never to 5 = always.

##### Engagement in PEBs at time 2

The respondents report how often they had engaged in the same seven behaviors during the last four weeks on a scale from 1 = never to 5 = always.

### Quantification and statistical analysis

We used linear mixed modeling to investigate whether anticipated positive and negative affect reported at Time 1 could predict the level of engagement with PEBs reported at Time 2, which was our main dependent variable. The main model (Model 1 in Table 2) included fixed effects of anticipated positive and negative affect as well as random intercepts at both behavior and individual levels. The random intercepts provided estimates for the variation between individuals as well as different sustainable behaviors. We did not include random slopes as their inclusion led to convergence issues. In addition, we included age, gender, household income, and education as control variables. Next, we controlled for past PEB (engagement with PEBs reported at Time 1) and intention to engage in PEBs reported at Time 1 by including them as fixed effects in the model (Models 2a, 2b, and 3 in Table 2). In addition to the random intercepts, these models also included random slopes for intention and past PEB over behaviors, which provided estimates for the variation between different behaviors when it comes to the linear effect of intention and past PEB. Thus, the multilevel analysis enables us to estimate the overall effects of anticipated affect, intentions, and past PEB on future PEB while respecting the variation between individuals and different sustainable actions included in the study. All statistical analyses were performed with R 4.3.0. Linear mixed models were carried out using *lmer* function in *lme4* package.

In addition, we have carried out exploratory mediation analyses (not preregistered) to test the plausibility of the claim that anticipated affect could be both a consequence and an antecedent of continued sustainable behaviors (e.g.,^6^). We have tested a theoretical model in which pro-environmental engagement reported at Time 1 is associated with anticipation of more positive and less negative feelings in the future, which enhances both pro-environmental behavioral intentions and future pro-environmental engagement. Note that this test is not preregistered and is exploratory. We have carried out this analysis to merely test whether such a mechanism could account for variation in the observed data. The analysis was carried out with a structural equation modeling approach using *lavaan* package.
