# Supplementary material for: Positive anticipated affective reactions increase pro-environmental behavior
Source: iScience. 2025 Apr 8;28(5):112389. doi: 10.1016/j.isci.2025.112389 (PMC12049809; doi:10.1016/j.isci.2025.112389)
Supplement: Document S1. Tables S1–S3 [file mmc1.pdf]

iScience, Volume *28*

Supplemental information

Positive anticipated affective reactions increase pro-environmental behavior

Camilla Strömbäck, Per A. Andersson, Erkin Asutay, Hulda Karlsson-Larsson, and Daniel Västfjäll

Supplementary material

The first part of this supplementary material contains the survey in English, while the second part contains the original version in Swedish.

Additional analyses

Table S1. Standardized coefficient estimates for the fixed effects model predicting PEBs reported at Time 2, including warm-glow. * *p<0.05* ** *p<0.01* *** *p<0.001*

	Model 1		*Model 2*		*Model 3*	
Predictors	*std. Beta*	*standardized CI*	*std. Beta*	*standardized CI*	*std. Beta*	*standardized CI*
(Intercept)	-0.03	-0.37, 0.32	-0.00	-0.09, 0.08	-0.00	-0.08, 0.08
positive					0.05 ***	0.03, 0.07
negative					-0.04 ***	-0.06, -0.02
Age	0.05 ***	0.02, 0.07	0.01	-0.00, 0.03	0.01	-0.01, 0.03
Gender [2]	0.05	-0.00, 0.11	0.01	-0.03, 0.04	0.01	-0.03, 0.04
Gender [3]	-0.04	-0.50, 0.43	-0.09	-0.38, 0.21	-0.07	-0.36, 0.23
Education	0.04 **	0.02, 0.07	0.02	-0.00, 0.03	0.02	-0.00, 0.03
income	-0.05 ***	-0.08, -0.02	-0.03 **	-0.04, -0.01	-0.03 **	-0.04, -0.01
PEBT1			0.44 ***	0.41, 0.47	0.42 ***	0.39, 0.45
intention			0.34 ***	0.31, 0.37	0.31 ***	0.28, 0.34
warmglow	0.21 ***	0.18, 0.24	0.04 ***	0.02, 0.05	0.02 **	0.01, 0.04
Marginal R^2 / Conditional R^2	.05 / .33		.63 / .66		.64 / .67	

Table S2. Correlation matrix between positive affect for each individual behavior and reported behavior at T2

Variables	Pos. affect shower	Pos. affect public transportation	Pos. affect turn lights off	Pos. affect vegetarian	Pos. affect recycle	Pos. affect hang dry clothes	Pos. affect lower temp.
Shower	0.287	0.108	0.080	0.059	0.155	0.134	0.207
Public transportation	-0.006	0.417	0.159	0.251	0.122	0.068	0.116
Turn lights off	0.129	0.160	0.458	0.143	0.222	0.200	0.240
Vegetarian	0.052	0.301	0.150	0.640	0.190	0.122	0.129
Recycle	0.091	0.220	0.155	0.119	0.371	0.204	0.152
Hang dry clothes	0.096	0.083	0.107	0.082	0.133	0.410	0.158
Lower temperature	0.099	0.095	0.130	0.100	0.130	0.193	0.476

.

Table S3. Correlation matrix between negative affect for each individual behavior and self-reported behavior at T2.

Variables	Neg. affect shower	Neg. affect public transportation	Neg. affect turn lights off	Neg. affect vegetarian	Neg. affect recycle	Neg. affect hang dry clothes	Neg. affect lower temp.
Shower	-0.330	-0.042	-0.121	-0.052	-0.112	-0.118	-0.164
Public transportation	-0.033	-0.265	-0.093	-0.092	-0.132	-0.087	-0.167
Turn lights off	0.059	-0.041	-0.317	-0.191	-0.040	0.005	-0.086
Vegetarian	0.035	-0.028	-0.145	-0.500	-0.067	-0.024	-0.092
Recycle	-0.138	-0.206	-0.205	-0.185	-0.367	-0.212	-0.125
Hang dry clothes	-0.087	-0.086	-0.083	-0.121	-0.099	-0.372	-0.114
Lower temperature	-0.085	-0.066	-0.067	-0.060	-0.081	-0.124	-0.437

Survey in English

The following consent form was shown to participants at the participant recruitment site:

Welcome!

This survey is part of a research project at (University name here) where the purpose is to study how people think about energy saving and the climate. The survey takes on average 15 minutes to complete. On this page follows some general information about the study.

How the study proceeds

You will be asked to answer questions in a survey on energy saving and climate friendly behaviors, which will involve your willingness to enact these behaviors, your emotions towards these behaviors, and your motives for the behaviors. At the start of the survey, we will ask some general demographic questions about age, gender, education and household income. At some point in the survey there will be an "attention check" so that we know that you are reading the questions. Your task at that point is to answer in accordance with the instructions given.

What happens to my data?

Your data will be analyzed anonymously in accordance with the current laws (EU GDPR 679/2016). The data is used only for the purpose of research. It will not be possible to link your answers to your personal identity.

Information about the study results

The study results will be handled on an aggregate level and will be published in scientific journals and their related webpages. Before publication the only way to get information is to contact the researchers at: (email here)

Compensation and risks

You will be compensated by (company name here) for your participation if you conduct the survey. There are no known risks in participating in this study.

Voluntary participation

Participation is voluntary and anonymous. At any point in time you may quit the study by shutting down this window.

The researcher responsible for this project

The researcher responsible for this project is (name of researcher and university here). If anything is unclear about your participation you may contact him/her at (email here)

Part 1 of the survey

Q1 Welcome!
This survey is part of a research project at [university name] aimed at studying how people think about energy saving and the climate. The survey takes approximately 15 minutes on average. The person responsible for this study and the project is [name] at [university name]. If anything is unclear regarding your participation, you can contact him at [email address].

- ○ I agree to participate in the study (1)
- ○ I do not agree to participate in the study (2)

Q1 What is your gender

- ○ Male (1)
- ○ Female (2)
- ○ Other/ do not want to tell (3)

Q2 How old are you?

____

Q3 What is your highest level of completed education?

- Less than ten years of compulsory schooling (1)
- 2-year upper secondary education or vocational school (2)
- 3- or 4-year upper secondary education (3)
- University or college education shorter than 3 years (4)
- University or college education for 3 years or more (5)

Q4 What is approximately your household's monthly income before taxes?

- Less than SEK 15 000 (1)
- SEK 15 000-24 999 (2)
- SEK 25 000-34 999 (3)
- SEK 35 000-44 999 (4)
- SEK 45 000-54 999 (5)
- SEK 55 000-64 999 (6)
- SEK 65 000-74 999 (7)
- SEK 75 000-84 999 (8)
- SEK 85 000-94 999 (9)
- SEK 95 000 or more (10)

Q11 How many people are in your household?

____

Q5 Do you, or someone else in your household, pay rent?

- No (1)
- Yes, I pay rent (2)
- Yes, I contribute financially to the rent (3)
- Yes, but I do not contribute financially to the rent (4)

Display This Question:

If Q5 != No

Q6 What is included in the rent?

- Heating (1)
- Warm water (2)
- Heating and warm water (3)
- Neither heating nor warm water (4)
- Do not know (5)

Q7 How carefully do you read the instructions on this page? This is an attention check, you shall answer ”not at all carefully” on this question.

- ◯ Very carefully (1)
- ◯ Carefulyl (2)
- ◯ Partly carefully (3)
- ◯ Not particularly carefully (4)
- ◯ Not at all carefully (5)

Q2 Think about paying your upcoming electricity bill. Indicate if you feel the emotions below, when you now think about paying this bill, by moving the cursor on the scale.

	Do not feel like this at all ... Feel very strongly like this<br>0 10 20 30 40 50 60 70 80 90 100
Anger ()	
Frustration ()	
Hopefullness ()	
Optimism ()	
Hopelessness ()	
Despair ()	
Anxiety ()	
Worry ()	

Q55 If you have any other emotion when you think about paying your upcoming electricity bill, you can indicate it below. (this question is optional)

	Do not feel like this at all										Feel very strongly like this
	0	10	20	30	40	50	60	70	80	90	100
Other emotion (you can indicate which one) ()											

Page Break

Q12 Think about climate change. For each word, move the cursor to indicate how you feel when you think about climate change.

	Do not feel like this at all									Feel very strongly like this
	0	10	20	30	40	50	60	70	80	90	100
Anger()											
Frustration ()											
Hoppfullness()											
Optimism ()											
Hopelessness ()											
Despair ()											
Anxiety ()											
Worry ()											

Q56 If you have any other emotion when you think about climate change, you can indicate it below. (this question is optional)

	Do not feel like this at all									Feel very strongly like this
	0	10	20	30	40	50	60	70	80	90	100
Other emotion (you can indicate which one) ()											

Page Break

Q25 Think about today's societal development in Sweden. For each word, move the cursor to indicate how you feel when you think about today's societal development in Sweden.

	Do not feel like this at all									Feel very strongly like this
	0	10	20	30	40	50	60	70	80	90	100
Anger ()											
Frustration ()											
Hopefulness ()											
Optimism ()											
Hopelessness ()											
Despair ()											
Anxiety ()											
Worry ()											

Q29 It is possible to feel both positive and negative emotions simultaneously about a behavior. Below are a number of questions where you should try to predict how you would feel when performing these behaviors.

Q30 How strong emotions do you think you would experience when you take a shower that last for less than 5 minutes?

	No emotions 0	Very strong emotions 100
Positive emotions ()		
Negative emotions ()		

Q37 How strong emotions do you think you would experience when you walk, bike, or use public transportation instead of driving?

	No emotions 0	Very strong emotions 100
Positive emotions ()		
Negative emotions ()		

Q36 How strong emotions do you think you would experience when you turn the lights off when leaving a room?

	No emotions 0	Very strong emotions 100
Positive emotions ()		
Negative emotions ()		

Q 35 How strong emotions do you think you would experience when you choose another source of protein than animal-based meat?

	No emotions	Very strong emotions
	0	100
Positive emotions ()		
Negative emotions ()		

---

35 How strong emotions do you think you would experience when you recycle household waste?

	No emotions	Very strong emotions
	0	100
Positive emotions ()		
Negative emotions ()		

Q33 How strong emotions do you think you would experience when you hang-dry clothes instead of tumble-drying them?

	No emotions	Very strong emotions
	0	100
Positive emotions ()		
Negative emotions ()		

Q38 How strong emotions do you think you would experience when you have an indoor temperature of 20 degrees Celsius or lower?

	No emotions	Very strong emotions
	0	100
Positive emotions ()		
Negative emotions ()		

Q13 The following questions are about whether you consciously avoid doing certain things. You will answer whether you agree or not. There can be various reasons why you avoid these things.

	Do not agree at all (1)	Do not agress (2)	Do partly not agree (3)	Agree to some extent (4)	Agree (5)	Completely agree (6)
I sometimes avoid taking a shower, even though I would like to take one (1)	○	○	○	○	○	○
Sometimes I avoid washing clothes and wear them even though I would like to wear freshly washed clothes (2)	○	○	○	○	○	○
Sometimes I avoid turning on electrical appliances that I actually want to use (3)	○	○	○	○	○	○
Sometimes I avoid raising the heat in certain rooms, even though I would like to (4)	○	○	○	○	○	○

Sometimes I avoid turning on the lights, even though I would like to have them on (5)	○	○	○	○	○	○

Q14	Do not agree at all (1)	Do not agree (2)	Do partly not agree (3)	Agree to some extent (4)	Agree (5)	Completely agree (6)
Sometimes I avoid trips that require flying, even though I would like to travel (1)	○	○	○	○	○	○
Sometimes I avoid trips that require driving, even though I would like to travel (2)	○	○	○	○	○	○
Sometimes I avoid buying new clothes, even though I would like to have them (3)	○	○	○	○	○	○
Sometimes I avoid buying plastic bags, even when it would be more practical to have them (4)	○	○	○	○	○	○
Sometimes I avoid buying meat, even though I like eating it (5)	○	○	○	○	○	○

Q51 If you want, you can write here why you avoid certain behaviors mentioned above. (This question is entirely voluntary)

Q15 For the following questions, answer whether the statement is true about you or not

	Do not agree at all (1)	Do not agree (2)	Do partly not agree (3)	Agree to some extent (4)	Agree (5)	Completely agree (6)	Do not agree at all (7)
I do **not** want to know my current electricity price. (1)							
Regardless, I want to know the current electricity price. (2)							
I do **not** want to know what measures I can take to save electricity (3)							
Regardless, I want to know what measures I can take to save electricity (4)							
I do **not** want to know whether the measures I take to save electricity are effective. (5)							

Regardless, I want to know whether the measures I take to save electricity are effective. (6)	○	○	○	○	○	○	○
I do **not** want to know what measures others take to save energy (7)	○	○	○	○	○	○	○
Regardless, I want to know what measures others take to save energy. (8)	○	○	○	○	○	○	○

Q47 To what extent do you think you will engage in the following behaviors **during the next month**?

	1. Never (1)	2. Rarely (2)	3. Sometimes (3)	4. Often (4)	5. Always (5)
Take showers that last for less than 5 minutes (1)	○	○	○	○	○
Walk, bike, or use public transportation instead of driving (2)	○	○	○	○	○
Turn the lights off when leaving a room (3)	○	○	○	○	○
Choose another source of protein than animal-based meat (4)	○	○	○	○	○
Recycle household waste (5)	○	○	○	○	○
Hang-dry clothes instead of tumble-drying them (6)	○	○	○	○	○
Have an indoor temperature of 20 degrees Celsius or lower (7)	○	○	○	○	○

Q55 Below are a number of questions about electricity and energy. If you don’t know the correct answer, you can guess one of the options.

Q1 What is the difference between power and energy?

- Power is a measure of what kind of energy can be used (1)
- Power is a measure of how much energy can be used at a given time (2)
- Power is a measure of the quality of energy use (3)

Q2 What is the difference between electricity and energy?

- Electricity is found in outlets. Energy is found in things like gasoline (1)
- None at all. They are two words for the same thing (2)
- Electricity is a form of energy. An energy carrier that can efficiently and flexibly transfer energy. (3)

Q3 Which option best describes a watt?

- A watt is a measure of how much current there is in a wire (1)
- A watt is a measure of how much excess heat a current-carrying product produces (2)
- A watt is a measure of how much work is done at a given moment (3)

Q4 Which option best describes a volt?

- A volt is a measure of how much current there is in a wire (1)
- A volt is a measure of electrical voltage (2)
- A volt is a measure of how dangerous electricity is (3)

Q5 Which type of power generates the most electricity in Sweden?

- Hydropower (1)
- Nuclear power (2)
- Wind power (3)
- Solar power (4)
- Oil (5)
- Biofuel (6)

Q6 When looking at the energy labeling of household products, which of the letters below indicates the most energy-efficient option?

- A (1)
- C (2)
- E (3)

Q7 From the categories below, choose the two that you think use the most energy in an average household over a year:

- ☐ Heating (1)
- ☐ Warm water (2)
- ☐ Household appliances in the bathroom (washing machine, dryer, etc.) (3)
- ☐ Household appliances in the kitchen (refrigerator, dishwasher, stove, oven, etc.) (4)
- ☐ Household appliances in the rest of the home (TV, computer, rechargeable devices, etc.) (5)
- ☐ Lighting (6)
- ☐ Ventilation (7)

Q8

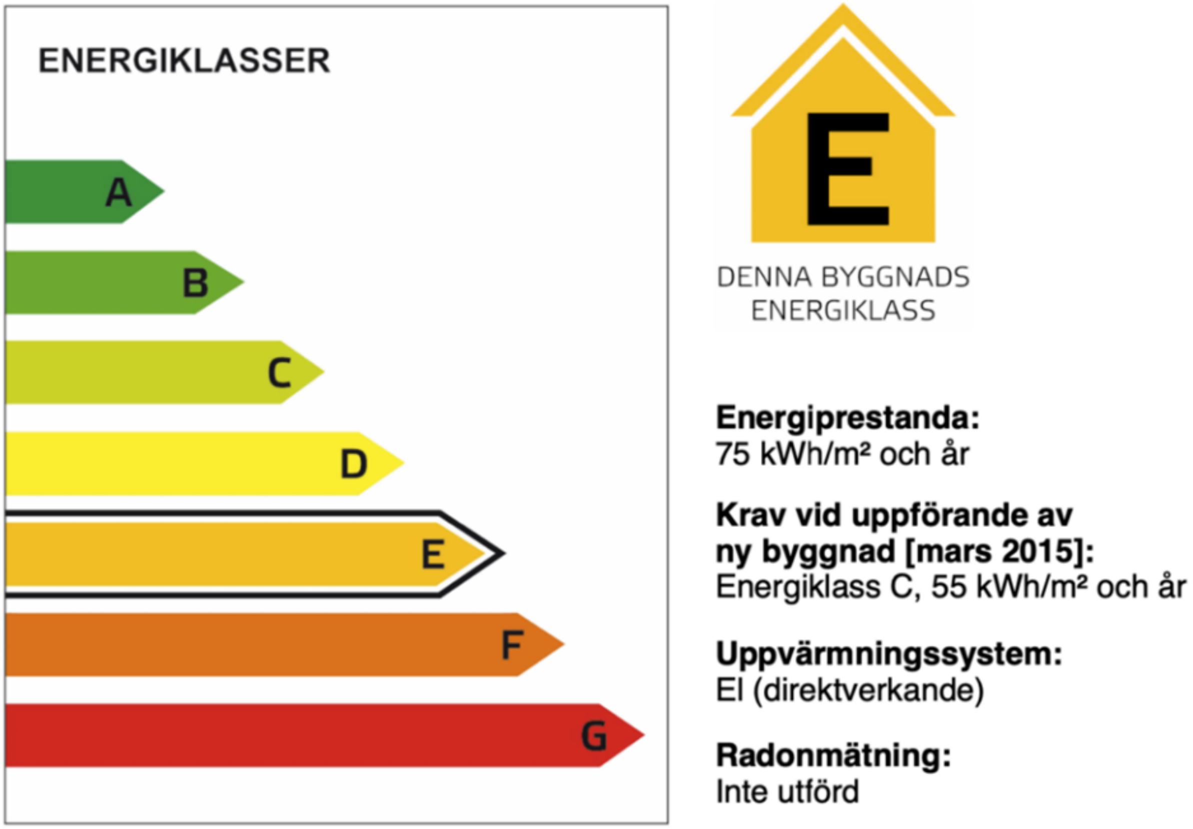

What can be inferred from the image above?

- ◯ Energy class shows how energy-efficient the house is in relation to its size (1)
- ◯ Energy class shows how much the house consumes at its peak consumption (2)
- ◯ A house with energy class E has low energy costs. (3)

Q9 Many households receive two or more electricity bills for the same time period, seemingly for the same electricity. What is the reason for this?

- One bill is for the electricity itself, while the other is for the transmission in the power line (1)
- One bill is for the energy and the other for the power (2)
- One is for the electricity you buy, the other is for taxes and VAT (3)

Q48 To what extent do you agree with the following statements?

	1. Do not agree at all (1)	2 (2)	3 (3)	4 (4)	5 (5)	6 (6)	7. Completely agree (7)
My household’s consumption and lifestyle impact environmental sustainability (1)	○	○	○	○	○	○	○
By reducing my consumption, I can contribute to the preservation of environmental sustainability (2)	○	○	○	○	○	○	○
If I act sustainably, it will encourage others to do the same (3)	○	○	○	○	○	○	○

Q50 To what extent do you agree with the following statements?

	1. do not agree (1)	2. do partly not agree (2)	3. neither agree nor disagree (3)	4. partly agree (4)	5. agree(5)
In most ways my life is close to my ideal. (1)	○	○	○	○	○
The conditions of my life are excellent. (2)	○	○	○	○	○
I am satisfied with my life (3)	○	○	○	○	○
So far I have gotten the important things I want in life (4)	○	○	○	○	○
If I could live my life over, I would change almost nothing (5)	○	○	○	○	○

Q1 Below are a number of statements about saving electricity this winter.

If any of these are not a reason for you, or if you do not save energy at all, you can answer 0, on the far left of the scale.

Q2 I save energy because I want to…

	Not at all a motive for me — Strong motive for me (0 10 20 30 40 50 60 70 80 90 100)
…have lower electricity costs ()	
…feel positive emotions after saving electricity ()	
…reduce climate and other environmental impacts ()	
... contribute to lower electricity prices and increased energy security for the whole society ()	
... contribute to lower electricity prices and increased energy security for the whole society ()	
…be independent of Russian energy ()	
other reason ()	

Q3 State any other reasons you may have for saving electricity (if you have any, this question is optional)

____

____

____

____

____

Q40 You should now indicate how often you have engaged in a number of behaviors **over the past four weeks.**

Q39 Taken showers that lasted for less than 5 minutes

- 1. Never (1)
- 2. Rarely (2)
- 3. Sometimes (3)
- 4. Often (4)
- 5. Always (5)

Q41 Walked, biked, or used public transportation instead of driving

- 1. Never (1)
- 2. Rarely (2)
- 3. Sometimes (3)
- 4. Often (4)
- 5. Every time the option has been available (5)

Q42 Turned the lights off when leaving a room

- 1. Never (1)
- 2. Rarely (2)
- 3. Sometimes (3)
- 4. Often (4)
- 5. Every time the option has been available (5)

Q43 Chosen another source of protein than animal-based meat

- 1. Never (1)
- 2. Rarely (2)
- 3. Sometimes (3)
- 4. Often (4)
- 5. Every time the option has been available (5)

Q44 Recycled household waste

- 1. Never (1)
- 2. Rarely (2)
- 3. Sometimes (3)
- 4. Often (4)
- 5. Always (5)

Q45 Hang-dried clothes instead of tumble-drying them

- 1. Never (1)
- 2. Rarely (2)
- 3. Sometimes (3)
- 4. Often (4)
- 5. Every time the option has been available (5)

Q46 Had an indoor temperature of 20 degrees Celsius or lower

- 1. Never (1)
- 2. Rarely (2)
- 3. Sometimes (3)
- 4. Often (4)
- 5. Always (5)

	Tired	Excited
How do you feel right now?		

	Dissatisfaction	Happiness
How do you feel right now?		

Q49 How well do these statements apply to you?

	1. Do not apply to me at all (1)	2 (2)	3 (3)	4 (4)	5 (5)	6 (6)	7. Apply completely to me (7)
I would feel good about myself if I took action to reduce climate change (1)	○	○	○	○	○	○	○
I would experience positive emotions if I did what I could to combat climate change (2)	○	○	○	○	○	○	○

Q28 How well do these statements apply to you?

	Disagree (1)	Partly disagree (2)	Neither disagree nor agree (3)	Partly agree (4)	Agree (5)
I might have difficulty paying the next electricity bill (1)	○	○	○	○	○
My financial situation is good (2)	○	○	○	○	○

Q54 Do you believe that climate change is caused by humans?

○ Yes (1)

○ Yes, partly (2)

○ No (3)

Part 2 (sent out approximately four weeks after part 1)

Q3 Welcome! This survey is part of a research project at [university name] aimed at studying how people think about energy saving and the climate. The survey takes approximately 5 minutes on average. The person responsible for this study and the project is [name] at [university name]. If anything is unclear regarding your participation, you can contact him at [email address].

○ I agree to participate in the study (1)

○ I do not agree to participate in the study (2)

Q1 You should now indicate how often you have engaged in a number of behaviors over the past four weeks.

Q2 Taken showers that lasted for less than 5 minutes

- 1. Never (1)
- 2. Rarely (2)
- 3. Sometimes (3)
- 4. Often (4)
- 5. Always (5)

Q4 Walked, biked, or used public transportation instead of driving

- 1. Never (1)
- 2. Rarely (2)
- 3. Sometimes (3)
- 4. Often (4)
- 5. Every time the option has been available (5)

Q5 Turned the lights off when leaving a room

- 1. Never (1)
- 2. Rarely (2)
- 3. Sometimes (3)
- 4. Often (4)
- 5. Every time the option has been available (5)

Q9 Chosen another source of protein than animal-based meat

- 1. Never (1)
- 2. Rarely (2)
- 3. Sometimes (3)
- 4. Often (4)
- 5. Every time the option has been available (5)

Q6 Recycled household waste

- 1. Never (1)
- 2. Rarely (2)
- 3. Sometimes (3)
- 4. Often (4)
- 5. Always (5)

Q7 Hang-dried clothes instead of tumble-drying them

- 1. Never (1)
- 2. Rarely (2)
- 3. Sometimes (3)
- 4. Often (4)
- 5. Every time the option has been available (5)

Q8 Had an indoor temperature of 20 degrees Celsius or lower

- 1. Never (1)
- 2. Rarely (2)
- 3. Sometimes (3)
- 4. Often (4)
- 5. Always (5)

Q22 Below are two questions about the behaviors mentioned above on this page

---

Q10 I believe that most of my friends engage in the climate-friendly behaviors mentioned above

- Disagree (1)
- Partly disagree (2)
- Neither disagree nor agree (3)
- Partly agree (4)
- Agree (5)

Q12 I believe that most of my friends think it is morally right to engage in the climate-friendly behaviors mentioned above

- Disagree (1)
- Partly disagree (2)
- Neither disagree nor agree (3)
- Partly agree (4)
- Agree (5)

Q17 The statements below are about seeking information. By seeking, we mean **actively looking for information**, which can include searching online, but also asking others or looking for other sources that are not on the internet. Which of the following behaviors have you engaged in during the past four weeks?

	Yes (1)	No (2)
I have searched for information about the electricity price (1)	○	○
I have searched for information about the measures I can take to save electricity (2)	○	○
I have searched for information about whether the measures I take to save electricity are effective (3)	○	○
I have searched for information about the measures others take to save energy (4)	○	○

Q18 The following questions are about whether you have tried to avoid thinking about certain things. Have you done any of the following in the past four weeks?

	Yes (1)	No (2)
I have tried to avoid thinking about my electricity bill (1)	○	○
I have tried to avoid thinking about the electricity price (2)	○	○
I have tried to avoid thinking about the measures I can take to save electricity (3)	○	○
I have tried to avoid thinking about whether the measures I take to save electricity are effective (4)	○	○
I have tried to avoid thinking about the measures others take to save energy (5)	○	○

Q13 How do you feel about having to read complicated information regarding how to save electricity at home?

	No emotions 0	Strong emotions 100
Positive emotions ()		
Negative emotions ()		

Display This Question:

If Q13 [Positive emotions] > 0

Q14 Since you answered that you had positive emotions about having to read complicated information regarding how to save electricity at home - can you put these emotions into words? Describe them below.

____

Display This Question:

If Q13 [Negative emotions] > 0

Q23 Since you answered that you had negative emotions about having to read complicated information regarding how to save electricity at home - can you put these emotions into words? Describe them below.

____

Q15

	Tired	Excited
How do you feel right now?		

Q16	Dissatisfaction	Happiness
How do you feel right now?		

Q19 To what extent do you agree with the following statements?

	1. do not agree (1)	2. do partly not agree (2)	3. neither agree nor disagree (3)	4. partly agree (4)	5. agree(5)
In most ways my life is close to my ideal. (1)	○	○	○	○	○
The conditions of my life are excellent. (2)	○	○	○	○	○
I am satisfied with my life (3)	○	○	○	○	○
So far I have gotten the important things I want in life (4)	○	○	○	○	○
If I could live my life over, I would change almost nothing (5)	○	○	○	○	○

Q21 I had difficulty paying my last electricity bill.

- ○ Does not apply at all (1)
- ○ Partly does not apply (2)
- ○ Neither does not apply nor does apply (3)
- ○ Partly does apply (4)
- ○ Does completely apply (5)

Q24 Here you can leave optional comments regarding the survey, if you have any comments.

____

Survey in Swedish

Följande information visades för deltagarna på PFM Researchs hemsida:

Välkommen!
Denna enkät ingår i ett forskningsprojekt vid Linköpings universitet där syftet är att studera hur människor tänker kring energisparande och klimatet. Enkäten tar ca 15 minuter i genomsnitt. På denna sida följer generell information om studien.

Hur studien går till

Du kommer att svara på frågor i en enkät om energisparande och klimatvänliga beteenden, som rör bland annat din vilja att utföra beteenden, dina känslor inför beteenden, och dina motiv till dessa. I början av enkäten ställer vi några allmänna demografiska frågor om ålder, kön, utbildning och hushållsinkomst. Någon gång under enkäten kommer en "uppmärksamhetsfråga" för att vi ska veta att du verkligen läser frågorna. Din uppgift är då att svara enligt instruktionen som ges.

Vad händer med mina uppgifter?

Dina uppgifter kommer att analyseras anonymt i enlighet med rådande lagstiftning (EU GDPR 679/2016). Uppgifterna används enbart i forskningssyfte. Det går inte att spåra dina svar till dina personuppgifter.

Information om studiens resultat

Studiens resultat kommer behandlas på aggregerad nivå, och kommer att publiceras i vetenskapliga tidskrifter och relaterade hemsidor. Innan publikation är enda sättet att få information att kontakta ansvarig forskare på: (email adress here)

Kompensation och risker

Du kommer att kompenseras av (company name here) för ditt deltagande om du utför enkäten. Det finns inga kända risker för att delta i denna studie.

Frivilligt deltagande

Deltagande är frivilligt och anonymt. Du kan när som helst avbryta studien genom att stänga detta fönster.

Ansvarig för forskningsprojektet

Ansvarig för denna studie och projektet är (name of researcher and university here). Om något är oklart kring ditt deltagande kan du kontakta honom på (email here)

Del 1 av enkäten

Q1 Välkommen!
Denna enkät ingår i ett forskningsprojekt vid [universitetets namn] där syftet är att studera hur människor tänker kring energisparande och klimatet. Enkäten tar ca 15 minuter i genomsnitt. Ansvarig för denna studie och projektet är [namn] vid [universitetets namn]. Om något är oklart kring ditt deltagande kan du kontakta honom på [mejladress]

- ◯ Jag samtycker till att delta i studien (1)
- ◯ Nej, jag vill inte delta i studien (2)

Q1 Vad är ditt kön?

- ◯ Man (1)
- ◯ Kvinna (2)
- ◯ Annat / vill inte uppge (3)

Q2 Hur gammal är du?

____

Q3 Vilken är din högsta avslutade utbildning?

- ◯ Ingen gymnasial utbildning (1)
- ◯ 2-årig gymnasieutbildning eller fackskola (2)
- ◯ 3- eller 4-årig gymnasieutbildning (3)
- ◯ Universitets- eller högskoleutbildning kortare än 3 år (4)
- ◯ Universitets- eller högskoleutbildning 3 år eller längre (5)

Q4 Hur stor är hushållets ungefärliga sammanlagda månadsinkomst före skatt?

- ○ Mindre än 15 000 kr (1)
- ○ 15 000-24 999 kr (2)
- ○ 25 000-34 999 kr (3)
- ○ 35 000-44 999 kr (4)
- ○ 45 000-54 999 kr (5)
- ○ 55 000-64 999 kr (6)
- ○ 65 000-74 999 kr (7)
- ○ 75 000-84 999 kr (8)
- ○ 85 000-94 999 kr (9)
- ○ 95 000 kr eller mer (10)

Q11 Hur många personer ingår i ditt hushåll?

____

Q5 Betalar du, eller någon i ditt hushåll, hyra för ert boende?

○ Nej (1)

○ Ja, jag betalar hyreskostnaden (2)

○ Ja, jag bidrar ekonomiskt till hyreskostnaden (3)

○ Ja, men jag bidrar inte ekonomiskt till hyreskostnaden (4)

Display This Question:

If Q5 != Nej

Q6 Vad inkluderas i hyran?

○ Uppvärmning (1)

○ Varmvatten (2)

○ Uppvärmning och varmvatten (3)

○ Varken uppvärmning eller varmvatten (4)

○ vet ej (5)

Q7 Hur noggrant läser du instruktionerna på denna sida? Detta är en uppmärksamhetskontroll, du ska svara "inte alls noggrant" på denna fråga.

- Mycket noggrant (1)
- Noggrant (2)
- Delvis noggrant (3)
- Inte särskilt noggrant (4)
- Inte alls noggrant (5)

Q2 Tänk på betalningen av din kommande elräkning. Indikera om du känner känslorna nedan, när du nu tänker på att betala denna elräkning, genom att flytta markören på skalan.

	Känner inte alls så										Känner mycket starkt så
	0	10	20	30	40	50	60	70	80	90	100
Ilska ()											
Frustration ()											
Hoppfullhet ()											
Optimism ()											
Hopplöshet ()											
Förtvivlan ()											
Ängslan ()											
Oro ()											

Q55 Om du har någon annan känsla när du tänker på att betala din kommande elräkning så kan du ange det här nedan. (denna fråga är frivillig)

	Känner inte alls så										Känner mycket starkt så
	0	10	20	30	40	50	60	70	80	90	100
Annan känsla (du kan ange vilken) ()											

Page Break

Q12 Tänk på klimatförändringen. För varje ord nedan, flytta markören för att indikera hur du känner dig, när du nu tänker på klimatförändringen.

	Känner inte alls så									Känner mycket starkt så
	0	10	20	30	40	50	60	70	80	90	100
Ilska ()											
Frustration ()											
Hoppfullhet ()											
Optimism ()											
Hopplöshet ()											
Förtvivlan ()											
Ängslan ()											
Oro ()											

Q56 Om du har någon annan känsla när du tänker på klimatförändringen så kan du ange det här nedan. (denna fråga är frivillig)

	Känner inte alls så									Känner mycket starkt så
	0	10	20	30	40	50	60	70	80	90	100
Annan känsla (du kan ange vilken) ()											

Page Break

Q25 Tänk på dagens samhällsutveckling i Sverige. För varje ord nedan, flytta markören för att indikera hur du känner dig, när du nu tänker på samhällsutvecklingen.

	Känner inte alls så									Känner mycket starkt så
	0	10	20	30	40	50	60	70	80	90	100
Ilska ()											
Frustration ()											
Hoppfullhet ()											
Optimism ()											
Hopplöshet ()											
Förtvivlan ()											
Ängslan ()											
Oro ()											

Q29 Det är möjligt att känna både positiva och negativa känslor samtidigt för ett beteende. Nedan följer ett antal frågor där du ska försöka förutspå hur du skulle känna vid utförandet av dessa beteenden.

Q30 Hur starka känslor tror du att du skulle känna i samband med att du tar en dusch som är maximalt 5 minuter?

	inga känslor<br>0	Mycket starka känslor<br>100
Positiva känslor ()		
Negativa känslor ()		

Q37 Hur starka känslor tror du att du skulle känna i samband med att du promenerar, cyklar eller åker kollektivt istället för att åka bil?

	inga känslor<br>0	Mycket starka känslor<br>100
Positiva känslor ()		
Negativa känslor ()		

Q36 Hur starka känslor tror du att du skulle känna i samband med att du släcker alla lampor när du lämnar ett rum?

	inga känslor<br>0	Mycket starka känslor<br>100
Positiva känslor ()		
Negativa känslor ()		

Q35 Hur starka känslor tror du att du skulle känna i samband med att du väljer en annan proteinkälla än animaliskt kött när du bestämmer vad du ska äta?

	inga känslor	Mycket starka känslor
	0	100
Positiva känslor ()		
Negativa känslor ()		

Q34 Hur starka känslor tror du att du skulle känna i samband med att du källsorterar ditt hushållsavfall?

	inga känslor	Mycket starka känslor
	0	100
Positiva känslor ()		
Negativa känslor ()		

Q33 Hur starka känslor tror du att du skulle känna i samband med att du hängtorkar tvätt istället för att använda torktumlare eller torkskåp?

	inga känslor	Mycket starka känslor
	0	100
Positiva känslor ()		
Negativa känslor ()		

Q38 Hur starka känslor tror du att du skulle känna i samband med att du har en inomhustemperatur på 20 grader eller kallare?

	inga känslor<br>0	Mycket starka känslor<br>100
Positiva känslor ()		
Negativa känslor ()		

Q13 De följande frågorna handlar om ifall du medvetet undviker att göra vissa saker. Du svarar ifall du håller med eller inte. Det kan finnas olika anledningar till varför du undviker dessa saker.

	Håller inte alls med (1)	Håller inte med (2)	Håller delvis inte med (3)	Håller delvis med (4)	Håller med (5)	Håller med helt (6)
Jag undviker ibland att ta en dusch, trots att jag skulle vilja ta en (1)						
Jag undviker ibland att tvätta kläder och använder dem trots att jag skulle vilja ha på mig nytvättade kläder (2)						
Jag undviker ibland att sätta på elektriska apparater, som jag egentligen vill använda (3)						
Jag undviker ibland att höja värmen i vissa rum, trots att jag skulle vilja det (4)						

Jag undviker ibland att tända lampor, trots att jag skulle vilja ha dem tända (5)	○	○	○	○	○	○

Q14	Håller inte alls med (1)	Håller inte med (2)	Håller delvis inte med (3)	Håller delvis med (4)	Håller med (5)	Håller med helt (6)
Jag undviker ibland resor som kräver att jag åker flyg, trots att jag skulle vilja resa (1)	○	○	○	○	○	○
Jag undviker ibland resor som kräver att jag åker bil, trots att jag skulle vilja resa (2)	○	○	○	○	○	○
Jag undviker ibland att köpa nya kläder, trots att jag skulle vilja ha dessa (3)	○	○	○	○	○	○
Jag undviker ibland att köpa plastpåsar, även när det skulle vara mer praktiskt att ha dem (4)	○	○	○	○	○	○

Jag undviker ibland att köpa kött, trots att jag gillar att äta det (5)	○	○	○	○	○	○

Q51 Om du vill, så kan du här skriva varför du undviker vissa beteenden ovan. (Denna fråga är helt frivillig)

____

Q15 På de följande frågorna svarar du ifall påståendet stämmer om dig eller inte	Håller inte alls med (1)	Håller inte med (2)	Håller delvis inte med (3)	Varken eller (4)	Håller delvis med (5)	Håller med (6)	Håller med helt (7)
Jag vill **inte** veta mitt nuvarande elpris. (1)	○	○	○	○	○	○	○
Oavsett vad, så vill jag veta nuvarande elpris. (2)	○	○	○	○	○	○	○
Jag vill **inte** veta vilka åtgärder jag kan göra för att spara el. (3)	○	○	○	○	○	○	○
Oavsett vad, så vill jag veta vilka åtgärder jag kan göra för att spara el. (4)	○	○	○	○	○	○	○
Jag vill **inte** veta huruvida åtgärderna jag gör för att spara el är effektiva. (5)	○	○	○	○	○	○	○

Oavsett vad, så vill jag veta huruvida åtgärderna jag gör för att spara el är effektiva. (6)	○	○	○	○	○	○	○
Jag vill **inte** veta vad för åtgärder andra gör för att spara energi. (7)	○	○	○	○	○	○	○
Oavsett vad, så vill jag veta vad för åtgärder andra gör för att spara energi. (8)	○	○	○	○	○	○	○

Q47 I vilken utsträckning tror du att du kommer att göra följande beteenden under **den kommande månaden**?

	1. Aldrig (1)	2. Sällan (2)	3. Ibland (3)	4. Oftast (4)	5. Alltid (5)
Duscha maximalt 5 minuter per duschtillfälle (1)	○	○	○	○	○
Promenera, cykla eller åka kollektivt istället för att åka bil (2)	○	○	○	○	○
Släcka alla lampor när du lämnar ett rum (3)	○	○	○	○	○
Välja en annan proteinkälla än animaliskt kött när du bestämmer vad du ska äta (4)	○	○	○	○	○
Källsortera ditt hushållsavfall (5)	○	○	○	○	○
Hängtorka tvätt istället för att använda torktumlare eller torkskåp (6)	○	○	○	○	○
Ha en inomhustemperatur på 20 grader eller kallare (7)	○	○	○	○	○

Q55 Här nedanför följer att antal frågor om el och energi. Om du inte vet rätt svar kan du gissa på ett av alternativen.

Q1 Vad är skillnaden mellan effekt och energi?

- ○ Effekt är ett mått på vilken slags energi som kan användas (1)
- ○ Effekt är ett mått på hur mycket energi som kan användas vid ett givet tillfälle (2)
- ○ Effekt är ett mått på energianvändningens kvalitet (3)

Q2 Vad är skillnaden mellan el och energi?

- ○ El finns i eluttag. Energi finns i t ex bensin (1)
- ○ Ingen alls. Det är två ord för samma sak (2)
- ○ El är en energiform. En energibärare som effektivt och flexibelt kan förflytta energi. (3)

Q3 Vilket alternativ beskriver bäst en watt?

- ○ En watt är ett mått på hur mycket ström det finns i en ledning (1)
- ○ En watt är ett mått på hur mycket överskottsvärme en strömförande produkt producerar (2)
- ○ En watt är ett mått på hur mycket arbete som utförs i en given stund (3)

Q4 Vilket alternativ beskriver bäst en volt?

- ○ En volt är ett mått på hur mycket ström det finns i en ledning (1)
- ○ En volt är ett mått på den elektriska spänningen (2)
- ○ En volt är ett mått på hur farlig elektriciteten är (3)

Q5 Vilket kraftslag framställer mest el i Sverige?

- ○ Vattenkraft (1)
- ○ Kärnkraft (2)
- ○ Vindkraft (3)
- ○ Solkraft (4)
- ○ Olja (5)
- ○ Biobränsle (6)

Q6 Om du tittar på energimärkning av hushållsprodukter, vilken av bokstäverna nedan anger det mest energieffektiva alternativet?

- ○ A (1)
- ○ C (2)
- ○ E (3)

Q7 Av nedanstående kategorier, välj de två som du tror använder mest energi i en genomsnittlig bostad under ett år:

- ☐ Uppvärmning (1)
- ☐ Varmvatten (2)
- ☐ Hushållsapparater i badrum (tvättmaskin, torktumlare etc) (3)
- ☐ Hushållsapparater i kök (kylskåp, diskmaskin, spis, ugn etc) (4)
- ☐ Hushållsapparater i övriga bostaden (TV, dator, laddningsbara enheter etc) (5)
- ☐ Belysning (6)
- ☐ Ventilation (7)

Q8

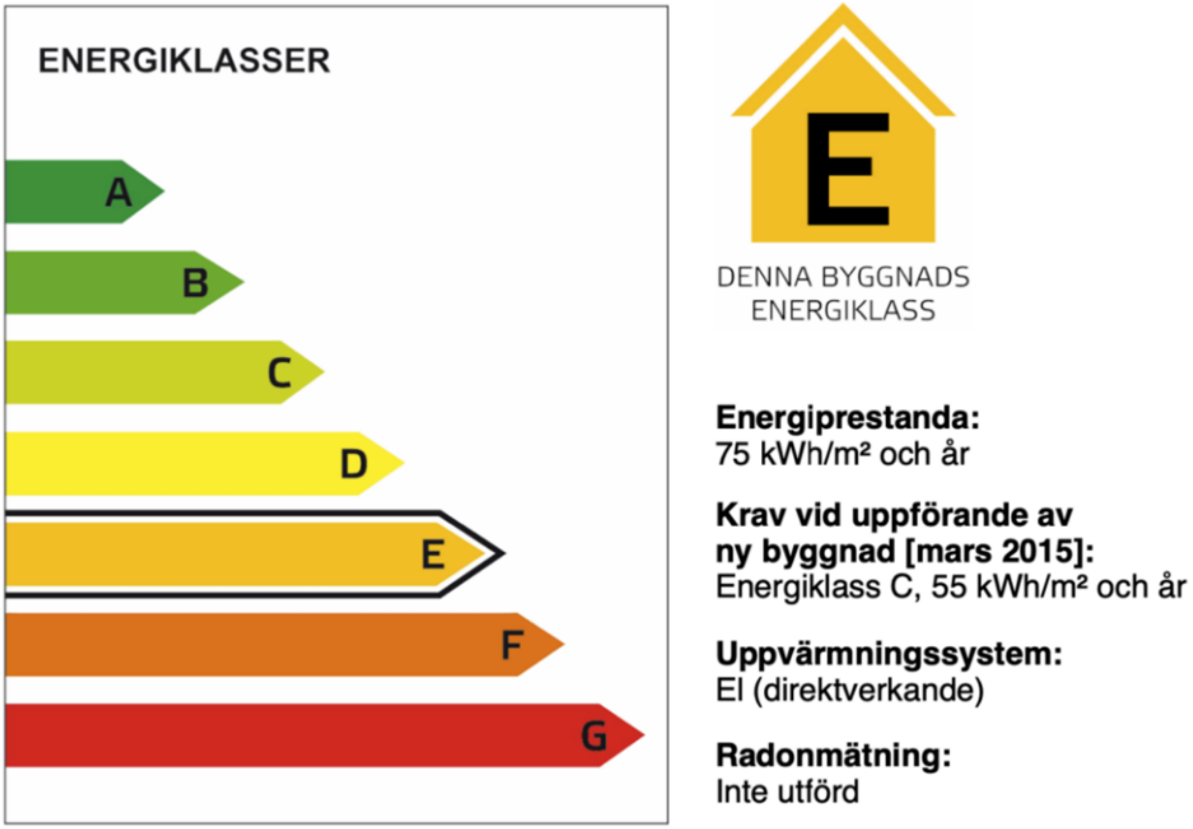

Vad kan man läsa ut av bilden ovan?

- ◯ Energiklass visar hur energieffektivt huset är i relation till storleken (1)
- ◯ Energiklass visar hur mycket huset förbrukar vid toppen av sin förbrukning (2)
- ◯ Ett hus med energiklass E har låga energikostnader. (3)

Q9 Många hushåll får två eller fler elräkningar för samma period, till synes för samma el. Vad beror det på?

- ○ Den ena räkningen avser själva elektriciteten, medan den andra avser frammatningen i elledningen (1)
- ○ Den ena räkningen avser energin och den andra effekten (2)
- ○ Den ena handlar om elen man köper, den andra om skatter och moms (3)

Q48 I vilken utsträckning håller du med om följande påståenden?

	1. Håller inte alls med (1)	2 (2)	3 (3)	4 (4)	5 (5)	6 (6)	7. Håller fullständigt med (7)
Mitt hushålls konsumtion och min livsstil påverkar miljömässig hållbarhet (1)	○	○	○	○	○	○	○
Genom att minska min konsumtion kan jag bidra till bevarandet av miljömässig hållbarhet (2)	○	○	○	○	○	○	○
Om jag agerar hållbart kommer det att uppmuntra andra att göra detsamma (3)	○	○	○	○	○	○	○

Q50 I vilken utsträckning instämmer du med om följande påståenden?

	1. instämmer inte (1)	2. instämmer delvis inte (2)	3. varken instämmer eller inte (3)	4. instämmer delvis (4)	5. instämmer (5)
Det mesta i mitt liv är nära mitt ideal (1)	○	○	○	○	○
Förutsättningarna för mitt liv är utmärkta (2)	○	○	○	○	○
Jag är nöjd med mitt liv (3)	○	○	○	○	○
Så här långt har jag fått de saker jag anser viktiga i livet (4)	○	○	○	○	○
Om jag kunde leva om mitt liv, skulle jag inte ändra någonting (5)	○	○	○	○	○

Q1 Nedanför följer ett antal påståenden om att spara el under denna vinter just nu.

Om något av dessa inte alls är ett skäl för dig, eller du inte alls sparar energi, så kan du svara 0, längst till vänster på skalan

Q2 Jag sparar el för att jag vill…

	Inte alls ett motiv för mig	Mycket starkt motiv för mig
	0 10 20 30 40 50	60 70 80 90 100
…ha lägre elkostnader ()		
…känna positiva känslor efter att ha sparat el ()		
…minska klimat- och annan miljöpåverkan ()		
...bidra till lägre elpriser och ökad energisäkerhet för hela samhället ()		
...bidra till lägre elpriser och ökad energisäkerhet för hela samhället ()		
…att vi ska vara oberoende av rysk energi ()		
annat skäl ()		

Q3 Uppge vilka andra skäl du eventuellt har till att spara el (om du har något, denna fråga är frivillig)

____

____

____

____

____

Q40 Du ska nu uppge hur ofta du gjort ett antal beteenden under de **senaste fyra veckorna**.

Q39 Duschat maximalt 5 minuter per duschtillfälle

- 1. Aldrig (1)
- 2. Sällan (2)
- 3. Ibland (3)
- 4. Oftast (4)
- 5. Alltid (5)

Q41 Promenerat, cyklat eller åkt kollektivt istället för att åka bil

- 1. Aldrig (1)
- 2. Sällan (2)
- 3. Ibland (3)
- 4. Oftast (4)
- 5. Varje gång alternativet har funnits (5)

Q42 Släckt alla lampor när du lämnat ett rum

- 1. Aldrig (1)
- 2. Sällan (2)
- 3. Ibland (3)
- 4. Oftast (4)
- 5. Varje gång alternativet har funnits (5)

Q43 Valt en annan proteinkälla än animaliskt kött när du bestämt vad du ska äta

- 1. Aldrig (1)
- 2. Sällan (2)
- 3. Ibland (3)
- 4. Oftast (4)
- 5. Varje gång alternativet har funnits (5)

Q44 Källsorterat ditt hushållsavfall

- 1. Aldrig (1)
- 2. Sällan (2)
- 3. Ibland (3)
- 4. Oftast (4)
- 5. Alltid (5)

Q45 Hängtorkat tvätt istället för att använda torktumlare eller torkskåp

- 1. Aldrig (1)
- 2. Sällan (2)
- 3. Ibland (3)
- 4. Oftast (4)
- 5. Varje gång alternativet har funnits (5)

Q46 Haft en inomhustemperatur på 20 grader eller kallare

- 1. Aldrig (1)
- 2. Sällan (2)
- 3. Ibland (3)
- 4. Oftast (4)
- 5. Alltid (5)

	Sömnig	Upprymd
Hur känner du dig just nu? ()		

	Missnöje	Glädje
Hur känner du dig just nu? ()		

Q49 Hur väl stämmer de här påståendena in på dig?

	1. Stämmer inte alls in på mig (1)	2 (2)	3 (3)	4 (4)	5 (5)	6 (6)	7. Stämmer helt och hållet in på mig (7)
Jag skulle känna mig duktig om jag agerade för att minska klimatförändringarna (1)	○	○	○	○	○	○	○
Jag skulle uppleva positiva känslor ifall jag gjorde vad jag kunde för att bekämpa klimatförändringar (2)	○	○	○	○	○	○	○

Q28 Hur väl stämmer de här påståendena in på dig?

	Håller inte med (1)	Håller delvis inte med (2)	Varken eller (3)	Håller delvis med (4)	Håller med (5)
Jag kommer kanske ha svårt att betala nästa elräkning. (1)	○	○	○	○	○
Min ekonomiska situation är god. (2)	○	○	○	○	○

Q54 Tror du att klimatförändringen är orsakad av människor?

○ Ja (1)

○ Ja, delvis (2)

○ Nej (3)

Del 2 (utskickad ca fyra veckor efter del 1)

Q3 Välkommen! Denna enkät ingår i ett forskningsprojekt vid [universitets namn] där syftet är att studera hur människor tänker kring energisparande och klimatet. Enkäten tar ca 5 minuter i genomsnitt. Ansvarig för denna studie och projektet är [namn] vid [universitetets namn]. Om något är oklart kring ditt deltagande kan du kontakta honom på [mejladress].

○ Ja, jag samtycker till att vara med i studien (1)

○ Nej, jag vill inte vara med i studien (2)

Q1 Hur ofta har du gjort följande beteende under de senaste 4 veckorna:

Q2 Duschat maximalt 5 minuter per duschtillfälle

- ◯ Aldrig (1)
- ◯ Sällan (2)
- ◯ Ibland (3)
- ◯ Ofta (4)
- ◯ Alltid (5)

Q4 Promenerat, cyklat eller åkt kollektivt istället för att åka bil

- ◯ Aldrig (1)
- ◯ Sällan (2)
- ◯ Ibland (3)
- ◯ Ofta (4)
- ◯ Varje gång alternativet har funnits (5)

Q5 Släckt alla lampor när du lämnat ett rum

- Aldrig (1)
- Sällan (2)
- Ibland (3)
- Ofta (4)
- Varje gång alternativet har funnits (5)

Q9 Valt en annan proteinkälla än animaliskt kött när du bestämt vad du ska äta

- Aldrig (1)
- Sällan (2)
- Ibland (3)
- Ofta (4)
- Varje gång alternativet har funnits (5)

Q6 Källsorterat ditt hushållsavfall

- Aldrig (1)
- Sällan (2)
- Ibland (3)
- Ofta (4)
- Alltid (5)

Q7 Hängtorkat tvätt istället för använt torktumlare eller torkskåp

- Aldrig (1)
- Sällan (2)
- Ibland (3)
- Ofta (4)
- Varje gång alternativet har funnits (5)

Q8 Haft en inomhustemperatur på 20 grader eller kallare

- Aldrig (1)
- Sällan (2)
- Ibland (3)
- Ofta (4)
- Alltid (5)

Q22 Här nedan följer två frågor om beteendena som nämnts ovanför på denna sida.

Q10 Jag tror att de flesta av mina nära bekanta sysslar med de klimatvänliga beteendena som nämnts ovan

- Håller inte med (1)
- Håller delvis inte med (2)
- Varken eller (3)
- Håller delvis med (4)
- Håller med (5)

Q12 Jag tror att de flesta av mina nära bekanta tycker att det är moraliskt rätt att utföra de klimatvänliga beteendena som nämnts ovan

- Håller inte med (1)
- Håller delvis inte med (2)
- Varken eller (3)
- Håller delvis med (4)
- Håller med (5)

Q17 Påståendena nedan handlar om att söka information. Med söka menar vi att **aktivt leta efter information**, vilket kan innebära att söka på nätet, men också att fråga andra eller leta efter andra källor som inte är internet. Vilka av följande beteenden har du utfört under de senaste fyra veckorna?

	Ja (1)	Nej (2)
Jag har sökt information om elpriset (1)	○	○
Jag har sökt information om de åtgärder jag kan göra för att spara el (2)	○	○
Jag har sökt information om huruvida åtgärderna jag gör för att spara el är effektiva (3)	○	○
Jag har sökt information om de åtgärder andra gör för att spara energi (4)	○	○

Q18 De följande frågorna handlar om ifall du försökt undvika att tänka på vissa saker. Har du gjort något av de följande under de senaste fyra veckorna?

	Ja (1)	Nej (2)
Jag har försökt undvika att tänka på min elräkning (1)	○	○
Jag har försökt undvika att tänka på elpriset (2)	○	○
Jag har försökt undvika att tänka på åtgärder jag kan göra för att spara el (3)	○	○
Jag har försökt undvika att tänka på huruvida åtgärderna jag gör för att spara el är effektiva (4)	○	○
Jag har försökt undvika att tänka på de åtgärder som andra gör för att spara energi (5)	○	○

Q13 Hur känner du inför att behöva läsa komplicerad information gällande hur man sparar el i hemmet?

	Inga känslor	Mycket starka känslor
	0	100
Positiva känslor ()		
Negativa känslor ()		

Display This Question:

If Q13 [Positiva känslor] > 0

Q14 Eftersom du svarade att du hade positiva känslor inför att behöva läsa komplicerad information gällande hur man sparar el i hemmet - kan du sätta ord på dessa känslor? Beskriv dem nedan.

____

Display This Question:

If Q13 [Negativa känslor] > 0

Q23 Eftersom du svarade att du hade negativa känslor inför att behöva läsa komplicerad information gällande hur man sparar el i hemmet - kan du sätta ord på dessa känslor? Beskriv dem nedan.

____

Q15

	Sömning	Upprymd
	0	100
Hur känner du dig just nu? ()		

Q16

	Missnöjd	Glad
	0	100
Hur känner du dig just nu? ()		

Q19 I vilken utsträckning instämmer du med följande påståenden?

	1. Instämmer inte (1)	2. Instämmer delvis inte (2)	3. Varken instämmer eller inte (3)	4. Instämmer delvis (4)	5. Instämmer (5)
Det mest i mitt liv är nära mitt ideal (1)	◯	◯	◯	◯	◯
Förutsättningarna för mitt liv är utmärkta (2)	◯	◯	◯	◯	◯
Jag är nöjd med mitt liv (3)	◯	◯	◯	◯	◯
Så här långt har jag fått de saker jag anser viktiga i livet (4)	◯	◯	◯	◯	◯
Om jag kunde leva om mitt liv, skulle jag inte ändra någonting (5)	◯	◯	◯	◯	◯

Q21 Jag hade svårt att betala min senaste elräkning

- Stämmer inte alls (1)
- Stämmer delvis inte (2)
- Varken stämmer eller inte stämmer (3)
- Stämmer delvis (4)
- Stämmer fullständigt (5)

Q24 Här kan du lämna frivilliga kommentarer angående enkäten, om du har några kommentarer.

____
